# 2373. "Longitudinal Investigation of Avidity Maturation following Multiple Doses of BNT162b2 mRNA Vaccine Including Bivalent Boosters among Nursing Home Residents and Healthcare Workers"

**DOI:** 10.1093/ofid/ofad500.1994

**Published:** 2023-11-27

**Authors:** Oladayo A Oyebanji, Vaishnavi Ragavapuram, Nicholas Sundheimer, Jürgen Bosch, Brigid Wilson, Stefan Gravenstein, Christopher King, David Canaday

**Affiliations:** Case Western Reserve University, Cleveland, Ohio; Case Western Reserve University, Cleveland, Ohio; Case Western Reserve University, Cleveland, Ohio; Case Western Reserve University, Cleveland, Ohio; VA Northeast Ohio Healthcare System, Cleveland, Ohio; Brown University, Providence, RI; Case Western Reserve University, Cleveland, Ohio; VA Northeast Ohio Healthcare System, Cleveland, Ohio

## Abstract

**Background:**

Increased breakthrough infections among nursing home residents (NHR) are clinically consequential despite good vaccine coverage and substantial antibody titers. While vaccination blunted severe outcomes, breakthrough infections may arise from lower vaccine-induced antibody quality. We aimed to assess vaccine-induced antibody quality among NHR and younger healthcare workers (HCW) using antibody avidity.

**Methods:**

We longitudinally sampled 50 NHR and 30 HCWs, with or without prior history of COVID-19, who received the BNT162b2 mRNA vaccine at 0-14 days before, 2 weeks, and 4-6 months after each vaccine dose up to the bivalent (BV) boosters. Primary outcomes included anti-spike, anti-Receptor Binding Domain (RBD), and avidity levels to the ancestral Wuhan, Delta, and Omicron BA.1 strains determined by bead-multiplex immunoassay and ability to bind after 6M urea vs. saline incubation.

**Results:**

After the primary series, anti-spike and RBD titers increased significantly to all 3 strains in both NHR and HCW. Avidity levels progressively increased across all time points up to the 3rd dose. Interestingly, this increase continued 6-8 months after vaccination suggesting that affinity maturation continues to occur for many months after the primary vaccination series. NHRs without prior infection have the poorest avidity levels to Delta and Omicron, especially before the 3rd dose. The 3rd dose raised avidity levels substantially in all groups and for all strains. Avidity levels after boosting, across the 3rd and 4th doses, demonstrate evidence of durability. Preliminary findings after BV boost show a continuation of avidity maturation with a larger impact on prior infected individuals.

Wuhan Spike Avidity

**Figure:**
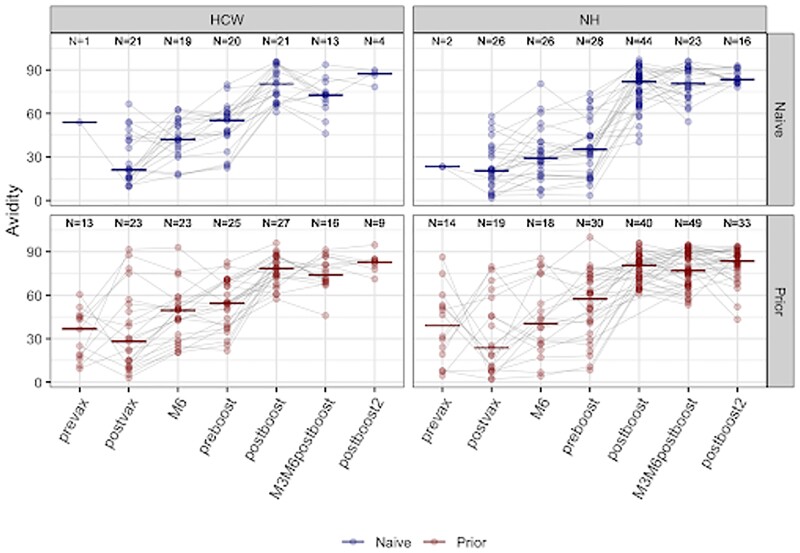

Avidity index of the Wuhan spike antibody across different time points among naive and prior infected individuals. Prevax- before primary series; Postvax- 2 weeks after primary series; M6- 6 months after primary series; Postboost- 2 weeks after 1st monovalent booster; M3M6postboost- 3-6months post booster; Postboost 2- 2 weeks after 2nd monovalent booster. NH- Nursing home; HCW- Healthcare Workers

Omicron BA.1 spike Avidity

**Figure:**
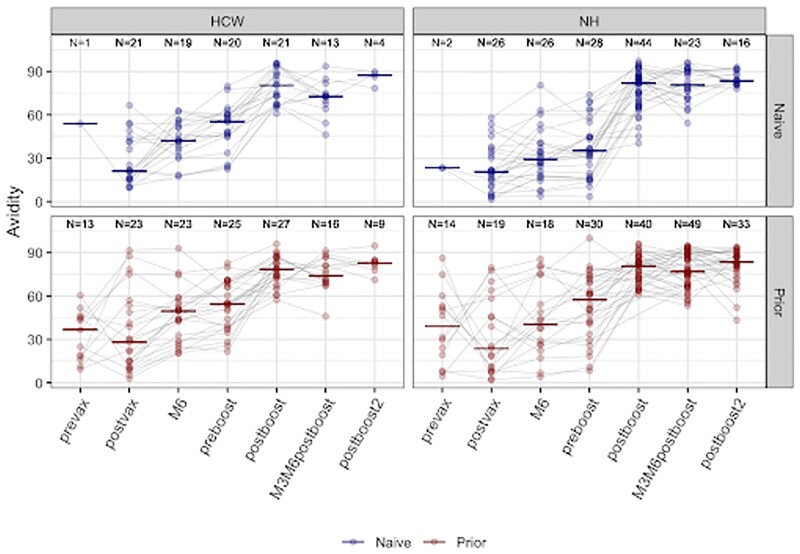

Avidity index of the BA.1 spike antibody across different time points among naive and prior infected individuals. Prevax- before primary series; Postvax- 2 weeks after primary series; M6- 6 months after primary series; Postboost- 2 weeks after 1st monovalent booster; M3M6postboost- 3-6months post booster; Postboost 2- 2 weeks after 2nd monovalent booster. NH- Nursing home; HCW- Healthcare Workers

**Conclusion:**

This study underscores the importance of booster vaccination among NHR and HCWs. The booster dose increases avidity, adding to vaccine-induced antibody functional ability. Higher avidity antibodies have higher cross-reactivity to other SARS-CoV-2 strains, as Wuhan boosting improves Delta and Omicron avidity, and should improve protection from ever-evolving strains. Higher avidities may help explain how the vaccine’s protective effects persist even while antibody titers fade between vaccine doses.

**Disclosures:**

**Stefan Gravenstein, MD, MPH**, CDC: Grant/Research Support|Genentech: Advisor/Consultant|Genentech: Grant/Research Support|GSK: Advisor/Consultant|GSK: Honoraria|Janssen: Advisor/Consultant|Janssen: Honoraria|NIH: Grant/Research Support|Pfizer: Grant/Research Support|Pfizer: Honoraria|Sanofi: Advisor/Consultant|Sanofi: Grant/Research Support|Sanofi: Honoraria|Seqirus: Grant/Research Support|Seqirus: Honoraria **David Canaday, MD**, Pfizer: Grant/Research Support

